# Korean Red Ginseng Attenuates Dysfunctions and Modulates Inflammatory and Neuroplasticity Markers in the Harmaline‐Induced Model of Essential Tremor

**DOI:** 10.1002/brb3.71470

**Published:** 2026-06-25

**Authors:** Monavareh Soti, Mehran Ilaghi, Leila Bagherzadeh, Fatemeh Shahsavari, Kristi A. Kohlmeier, Zeynab Pirmoradi, Mohammad Shabani

**Affiliations:** ^1^ Neuroscience Research Center, Institute of Neuropharmacology Kerman University of Medical Sciences Kerman Iran; ^2^ Department of Physical Medicine and Rehabilitation Northwestern University Feinberg School of Medicine Chicago Illinois USA; ^3^ Department of Drug Design and Pharmacology, Faculty of Health Sciences University of Copenhagen Copenhagen Denmark

**Keywords:** Cognition, Essential tremor, Harmaline, Korean red ginseng, Motor, *Panax*

## Abstract

**Background:**

Essential tremor (ET) is a prevalent movement disorder characterized by tremors and other motor and nonmotor symptoms which can significantly impact daily activities and quality of life. Current therapeutic options are limited in both efficacy and tolerability. Korean red Ginseng (*Panax*) has demonstrated neuroprotective properties in various neurological conditions, but its potential benefits in ET remain unexplored.

**Methods:**

ET was induced in male Swiss mice (*n* = 32) using Harmaline hydrochloride (10 mg/kg, intraperitoneal [i.p.]) administered on Days 1, 3, and 5. Korean red Ginseng (100 mg/kg) was given orally for 7 consecutive days. Motor function, anxiety‐like behaviors, and cognitive performance were assessed through behavioral testing. Gene expression analysis of Pro‐inflammatory mediators(TNF‐α, NF‐κB, IL‐1β, IL‐6) and neuroplasticity markers (Lingo‐1, NMDA) were performed in cerebellar tissue through RT‐qPCR.

**Results:**

Korean red Ginseng significantly attenuated Harmaline‐induced tremor severity and improved motor coordination on the rotarod test. Treatment also ameliorated anxiety‐like behaviors and fear‐associated memory deficits in Harmaline‐treated mice. At the molecular level, Korean red Ginseng suppressed Harmaline‐induced elevations in pro‐inflammatory markers (TNF‐α, NF‐κB, IL‐1β, IL‐6) and normalized the expression of Lingo‐1 and NMDA receptor genes in the cerebellum.

**Conclusions:**

Korean red Ginseng ameliorated both motor and nonmotor symptoms in an animal model of ET potentially through modulation of neuroinflammatory and neuroplasticity pathways. These findings suggest that Korean red Ginseng may represent a promising therapeutic option for ET management.

## Graphical abstract

Korean red Ginseng significantly reduces tremor severity, improves motor coordination, and ameliorates anxiety and cognitive deficits in a mouse model of essential tremor through suppression of neuroinflammatory markers and normalization of neuroplasticity genes in the cerebellum.

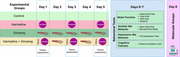

## 1 Introduction

Essential tremor (ET) is one of the most prevalent adult‐onset movement disorders, affecting over 1% of the general population and more than 5% of individuals over 65 years of age (Louis and McCreary 2021). ET is characterized by progressive postural and kinetic tremors primarily affecting the upper limbs, which significantly impacts quality of life by interfering with daily activities and social functioning (Gerbasi et al. 2022; Haubenberger and Hallett 2018). Despite its high prevalence and substantial burden on affected individuals, current therapeutic options for ET are largely restricted, provide only modest benefits, and are often accompanied by significant adverse effects, leading to frequent treatment discontinuation, which highlights the urgent need for more effective therapeutic alternatives.

Effective treatments should directly target the underlying disease mechanisms of ET. The pathophysiology of ET involves complex interactions between various neural circuits, with particular emphasis on cerebellar dysfunction (Hanajima et al. 2016). Growing evidence suggests that neuroinflammation plays a crucial role in ET pathogenesis (Mattson and Camandola 2001; Muruzheva, Ivleva, et al. 2022). Alterations of pro‐inflammatory cytokines, including tumor necrosis factor‐alpha (TNF‐α), interleukin‐1 beta (IL‐1β), and interleukin‐6 (IL‐6), have been implicated in cerebellar dysfunction and ET (Muruzheva, Traktirov et al. 2022; Pirmoradi, Esmaili, et al. 2025). These inflammatory mediators, regulated by the nuclear factor kappa B (NF‐κB) signaling pathway, can alter synaptic transmission and contribute to neuronal dysfunction (Mattson and Camandola 2001).

Neuroplasticity‐related proteins, particularly Lingo‐1 and NMDA receptors, have emerged as key players in ET. Lingo‐1, a negative regulator of neuronal survival and axonal regeneration, has been identified as a potential therapeutic target in ET (Agúndez et al. 2015; Pirmoradi, Esmaili, et al. 2025), with genetic studies revealing associations between Lingo‐1 variants and ET susceptibility (Clark et al. 2010; Vilariño‐Güell et al. 2010). Moreover, NMDA receptors are crucial mediators of synaptic plasticity and excitatory neurotransmission. Dysregulation of these neuroplasticity markers may contribute to abnormal cerebellar circuit function and tremor generation, suggesting that therapeutic strategies targeting these pathways could offer novel approaches for ET treatment (Piochon et al. 2010).

Korean red Ginseng, derived from *Panax Ginseng* Meyer, has demonstrated notable neuroprotective properties in various neurological conditions (Cho 2012; Lee et al. 2015), and the active compounds, particularly Ginsenosides, exhibit anti‐inflammatory and neuroplasticity‐enhancing effects (Baek et al. 2016; Bui et al. 2022; Chou et al. 2023). However, its potential therapeutic benefits in ET remain largely unexplored. Accordingly, we investigated the potential of Korean red Ginseng in the harmaline mouse model of ET, which has been widely used for investigating therapeutic interventions and neuroprotective strategies for ET. Harmaline, a β‐carboline alkaloid, induces tremors by acting as a potent tremorgenic agent and primarily disrupts the rhythmic activity of inferior olivary neurons (Lang and Handforth 2022; Zhan and Graf 2012). This results in synchronized oscillatory firing patterns within the olivocerebellar circuit, manifesting as action tremors that closely resemble those observed in human ET (Abbassian et al. 2024). Using the Harmaline‐induced model of ET, we examined the impact of Korean red Ginseng on motor function, anxiety‐like behavior, and cognitive performance. Additionally, we explored potential underlying molecular mechanisms of effects of Korean red Ginseng by analyzing expression of key inflammatory mediators and neuroplasticity‐related genes in cerebellar tissue.

## 2 Methods

### 2.1 Animals

Male Swiss mice (*n* = 32), aged 8–10 weeks and weighing between 20–25 g, were acquired from Kerman University of Medical Sciences for this study. The animals were housed under controlled conditions with ambient temperature maintained at 22°C ± 1°C and exposed to a fixed 12‐h light/dark schedule. Throughout the experimental period, mice had unrestricted access to food and water. To minimize stress during behavioral testing, the animals underwent a 2‐week adaptation period that included daily handling procedures. The experimental protocols implemented in this study were conducted in compliance with institutional standards and received approval from Kerman University of Medical Sciences Ethics Committee (IR.KMU.AEC.1403.038). This study was designed, conducted, and reported in accordance with the ARRIVE (Animal Research: Reporting of In Vivo Experiments) guidelines (Percie du Sert et al. 2020).

### 2.2 Experimental Design

The experimental subjects were divided into four groups through simple randomization, with eight mice per group:

Group 1 (Control): These animals served as the control group and underwent no treatment interventions.

Group 2 (Harmaline): To induce ET, mice received intraperitoneal (i.p.) injections of Harmaline hydrochloride dihydrate (Sigma, USA; cat. no. H1392) at 10 mg/kg on experimental Days 1, 3, and 5, following established protocols (Pirmoradi et al. 2024).

Group 3 (Ginseng): Animals received daily oral administration of Korean red Ginseng at 100 mg/kg for a continuous 7‐day period. The preparation was derived from 250 mg capsules containing powdered *Panax Ginseng* rhizome, standardized to contain 6.3–7.7 mg of total Ginsenosides expressed as Rg1. The product was supplied by Gol Daru Company (Tehran, Iran).

Group 4 (Harmaline + Ginseng): This group underwent both Harmaline injections (as described for Group 2) and daily Korean red inseng administration (as in Group 3). On days when both treatments were scheduled, Korean red Ginseng was administered orally 1 h prior to the Harmaline injection.

**FIGURE 1:**
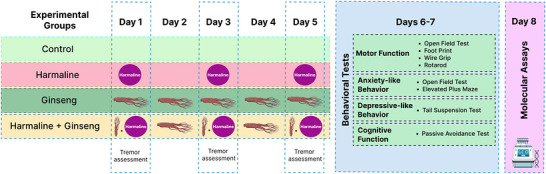
An overview of the timeline and experimental design of the study.

An illustration of the experimental design of the study is presented in Figure 1.

### 2.3 Behavioral Tests

A series of behavioral assessments was conducted to examine motor functioning, anxiety‐related behaviors, depressive‐like symptoms, and cognitive performance. The outcome assessor was blinded to group allocations.

#### 2.3.1 Assessment of Motor Function

##### 2.3.1.1 Tremor Scoring

Tremor evaluation was conducted using a quantitative assessment of tremor severity through a standardized five‐point rating scale as follows—Score 0: no detectable tremors, Score 1: sporadic tremors localized to head and neck regions, Score 2: periodic tremors affecting the entire body, Score 3: continuous whole‐body tremors, and Score 4: severe tremors resulting in impaired locomotion and postural instability (Mohammadi et al. 2019).

##### 2.3.1.2 Open Field Test

Locomotor activity was evaluated using a square arena (90 × 90 × 30 cm). Prior to testing, each subject underwent a 60‐min habituation period in the testing environment. Testing commenced with the placement of individual mice at the arena's center point. The assessment quantified several behavioral parameters, including total distance moved, total duration of movement, and mean locomotor velocity. Test sessions were recorded via an overhead camera system mounted 1.5 m above the apparatus, providing coverage of the testing field. Each trial lasted 5 min, with cleaning of the apparatus between subjects to eliminate olfactory cues. Behavioral analysis was conducted using Ethovision software (Noldus Information Technology, Wageningen, The Netherlands; version 7.1) (Gould et al. 2009).

##### 2.3.1.3 Foot Print Test

Gait characteristics were evaluated through analysis of footprint patterns. This assessment involved coating the posterior paws with innocuous paint followed by allowing mice to walk along a paper‐covered corridor at their spontaneous walking speed. The footprint impressions were analyzed to quantify two key parameters: stride width (the lateral separation between contralateral paw prints) and stride length (the longitudinal distance between successive right and left hind paw placements) (Abbassian et al. 2016).

##### 2.3.1.4 Wire Grip Test

Muscular strength was evaluated using a wire grip paradigm. The testing apparatus consisted of a horizontally mounted steel rod (80 cm length, 7 mm diameter). Each subject was suspended in a vertical orientation, allowing forelimb grasp of the rod. Following confirmation of secure grip, latency to fall was recorded. The protocol comprised three independent trials per subject, with 30‐min intertrial intervals to minimize fatigue effects. Trial sequence was randomized across subjects (Ranjbar et al. 2024).

##### 2.3.1.5 Rotarod

Motor coordination, balance, and endurance were assessed using an automated rotarod system (Hugo Sachs Electronik, Germany). The apparatus was programmed to accelerate linearly from 10 to 60 revolutions per minute. Testing consisted of three independent trials per subject, with each trial limited to a maximum duration of 300 s. A standardized 30‐min recovery interval was implemented between successive trials to prevent fatigue effects. Trial termination occurred upon either subject fall or completion of the maximum time period. The primary outcome measure was latency to fall, which quantified the duration of successful performance on the accelerating rod (Ranjbar et al. 2022).

#### 2.3.2 Assessment of Anxiety‐Like Behaviors

##### 2.3.2.1 Open Field Test

In addition to monitoring motor activity, the open field paradigm was utilized to evaluate anxiety‐related behavioral indices. Multiple parameters were quantified, including duration of time spent in the central zone, number of rearing, and grooming behavior (Kraeuter et al. 2019).

##### 2.3.2.2 Elevated Plus Maze

Anxiety‐related behaviors were evaluated using the elevated plus maze paradigm. The apparatus consisted of a cruciform platform with two open and two enclosed arms elevated from ground level. Each trial was initiated by positioning the subject at the central intersection, facing an open arm. Behavioral assessment was conducted over a 5‐min period, during which the duration of open arm exploration and entry frequency into both open and enclosed arms were assessed (Walf and Frye 2007).

#### 2.3.3 Assessment of Depressive‐Like Behaviors

##### 2.3.3.1 Tail Suspension Test

Depressive‐like behavior was assessed using the tail suspension paradigm. The protocol involved vertical suspension of individual subjects within a testing apparatus for a total duration of 6 min. The primary outcome measure was duration of immobility, which served as an index of behavioral despair (Can et al. 2012).

#### 2.3.4 Assessment of Learning and Memory

##### 2.3.4.1 Passive Avoidance Test

Fear‐associated learning and memory were evaluated using a two‐compartment passive avoidance system, comprising illuminated and darkened chambers connected by a guillotine‐type door. The protocol consisted of two distinct phases. During the training phase, subjects were initially positioned in the illuminated compartment for a 60‐s acclimation period. Following the opening of the door, subjects were permitted to transition to the darkened chamber based on innate preference. Upon entry, a brief electrical footshock (0.5 mA, 3‐s duration) was delivered via the floor grid. The number of shock presentations required for learning was recorded. Memory retention was evaluated following a 30‐min interval. The primary outcome measure was step‐through latency, with a maximum duration of 300 s. Additional performance indices included time spent in the dark compartment and number of dark compartment entries (Nazeri et al. 2016).

### 2.4 Molecular Assays

After behavioral assessment, subjects were euthanized via rapid decapitation, followed by immediate cerebellar extraction. Molecular analyses were performed on cerebellar tissue from four randomly selected animals per experimental group. This sample size is consistent with prior RT‐qPCR–based studies in Harmaline‐induced models of ET and was chosen to provide sufficient sensitivity for detecting group‐level changes while balancing ethical considerations and resource constraints (Pirmoradi, Ilaghi et al. 2025; Pirmoradi et al. 2024). The isolated tissue was frozen using dry ice and stored at −80°C until molecular processing. For transcriptional analysis, total RNA was extracted from cerebellar tissue of four randomly selected subjects per experimental group using Trizol reagent (Zaver Zist Azma, Iran). RNA integrity and quantification were assessed via nanodrop spectrophotometry (Thermo Scientific, USA). Subsequently, reverse transcription was performed using the Easy cDNA Synthesis Kit (Pars Tous, Iran) following manufacturer specifications. Transcriptional profiles were analyzed using real‐time quantitative PCR with SYBR‐Green chemistry on a Qiagen detection platform (Qiagen, Germany). The analysis encompassed inflammatory mediators (TNF‐α, NF‐κB, IL1‐β, IL‐6) as well as Lingo‐1 and NMDA. GAPDH served as the endogenous reference gene for expression normalization. Amplification was conducted using Real Q Plus 2× Master Mix (Pars Tous, Iran). Standard curves with serial cDNA dilutions were employed to validate amplification efficiency and linear dynamic range. Relative expression levels were calculated using the 2^−ΔΔCT^ methodology.

### 2.5 Statistical Analysis

Data analysis was conducted using GraphPad Prism software (version 8). Results were expressed as mean values with standard error of the mean (SEM). Normality of data distribution was assessed through both visual inspection of Q–Q plots and the Kolmogorov–Smirnov test. For normally distributed datasets, between‐group comparisons were conducted using one‐way or two‐way analysis of variance (ANOVA) followed by Tukey's post hoc analysis. Nonparametric data were evaluated using the Kruskal–Wallis test with subsequent Dunn's multiple comparison procedure. Statistical significance was established at *p* < 0.05.

## 3 Results

### 3.1 Korean Red Ginseng Attenuated Harmaline‐Induced Tremor Severity

**FIGURE 2:**
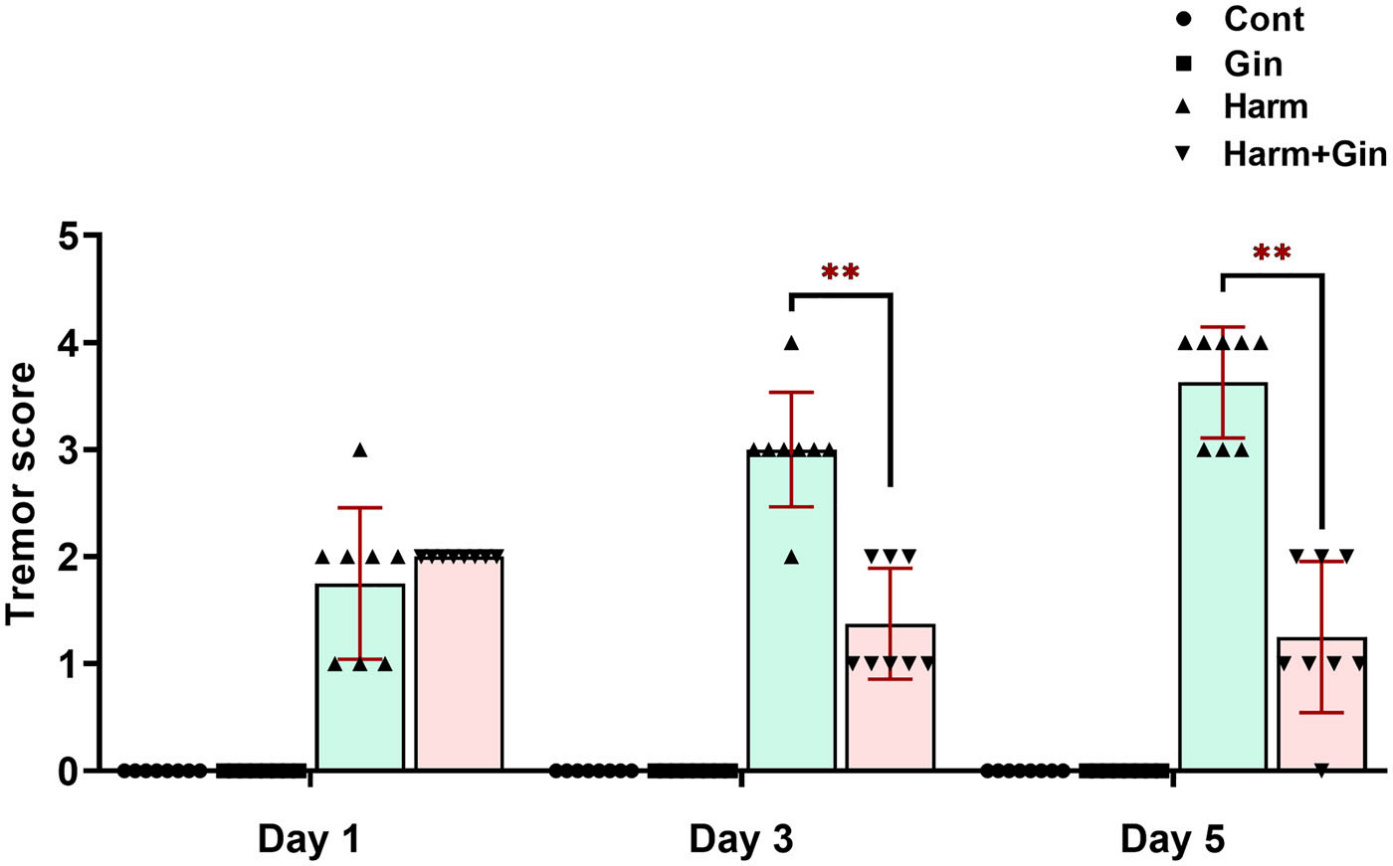
Assessment of tremor scores across experimental groups on Days 1, 3, and 5 of the experiment. Data are expressed as mean ± SEM. Statistical analysis revealed significantly higher tremor scores in Harmaline and Harmaline + Ginseng groups compared to both control and Ginseng groups across all time points (*p* < 0.0001, indicators omitted for clarity). A significant reduction was observed in tremor severity in the Harmaline + Ginseng group compared to the Harmaline group on Days 3 and 5 (**p < 0.01). Cont, control group; Gin, Ginseng group; Harm, Harmaline group; Harm + Gin, Harmaline + Ginseng group.

Harmaline administration induced significant tremors in experimental subjects on Days 1, 3, and 5 compared to the control group. Importantly, the Harmaline + Ginseng group showed markedly reduced tremor intensity compared to the Harmaline group on Days 3 and 5 (*p* < 0.01) (Figure 2), suggesting that treatment with Korean red Ginseng attenuated Harmaline‐induced tremor.

### 3.2 Effects of Harmaline and Korean Red Ginseng on Motor Parameters of Open Field Test

**FIGURE 3:**
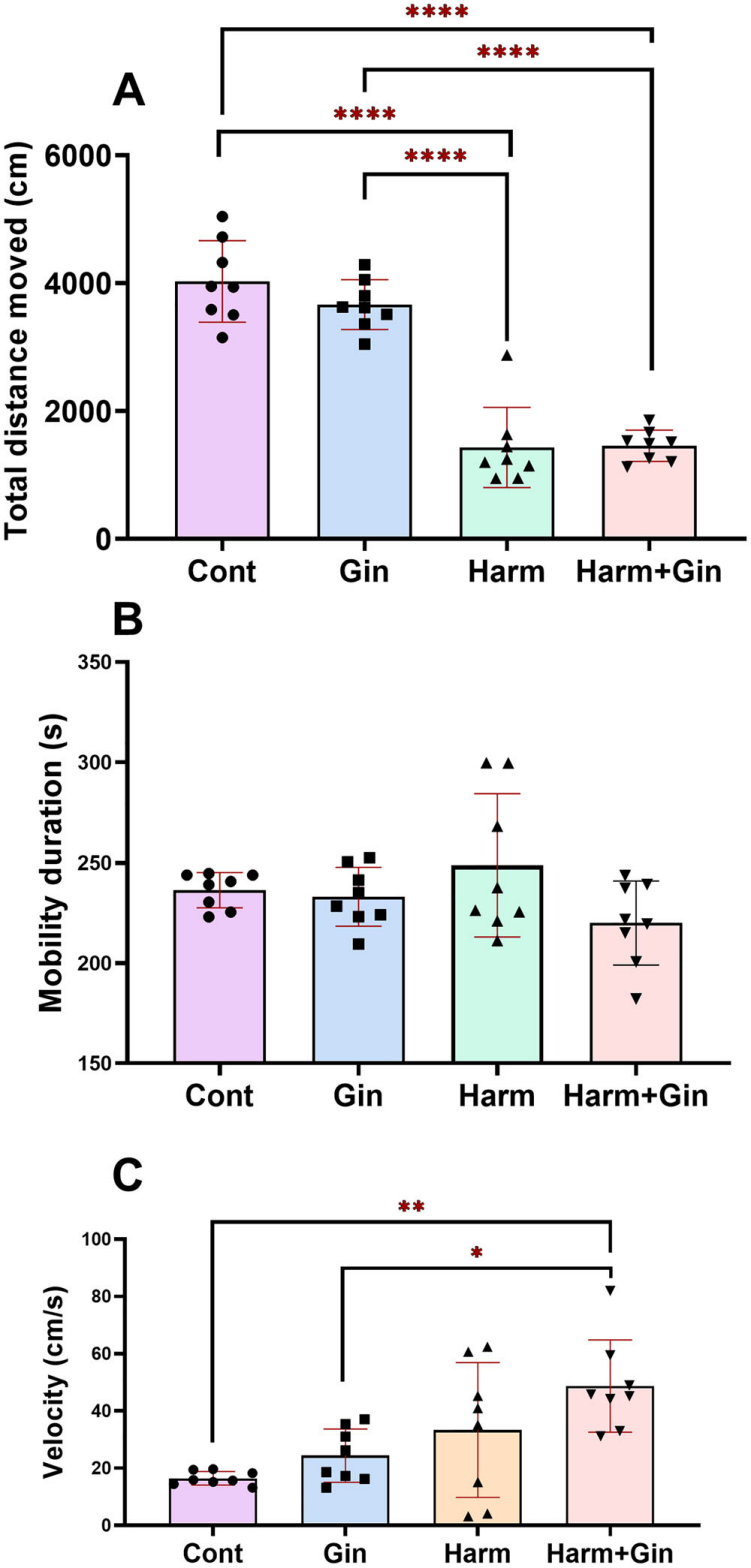
Open field assessment of locomotor activity across experimental groups. (A) Total distance moved, (B) mobility duration, and (C) velocity measurements. Data are expressed as mean ± SEM. *p < 0.05, **p < 0.01, ****p < 0.0001.

Analyzing the motor parameters in the open field test demonstrated that the Harmaline group showed marked reductions in total distance moved compared to both control and Ginseng‐only groups (*p* < 0.0001) (Figure 3A), but Harmaline treatment did not significantly affect the mobility duration (Figure 3B) and velocity (Figure 3C). Treatment with Korean red Ginseng did not attenuate the deficits in total distance moved caused by Harmaline (Figure 3A). Mobility duration was also not affected (Figure 3B); however, the velocity was significantly higher than that seen in the Ginseng‐only groups (*p* < 0.05) (Figure 3C).

### 3.3 Effects of Korean Red Ginseng on Motor Coordination and Muscle Strength

**FIGURE 4:**
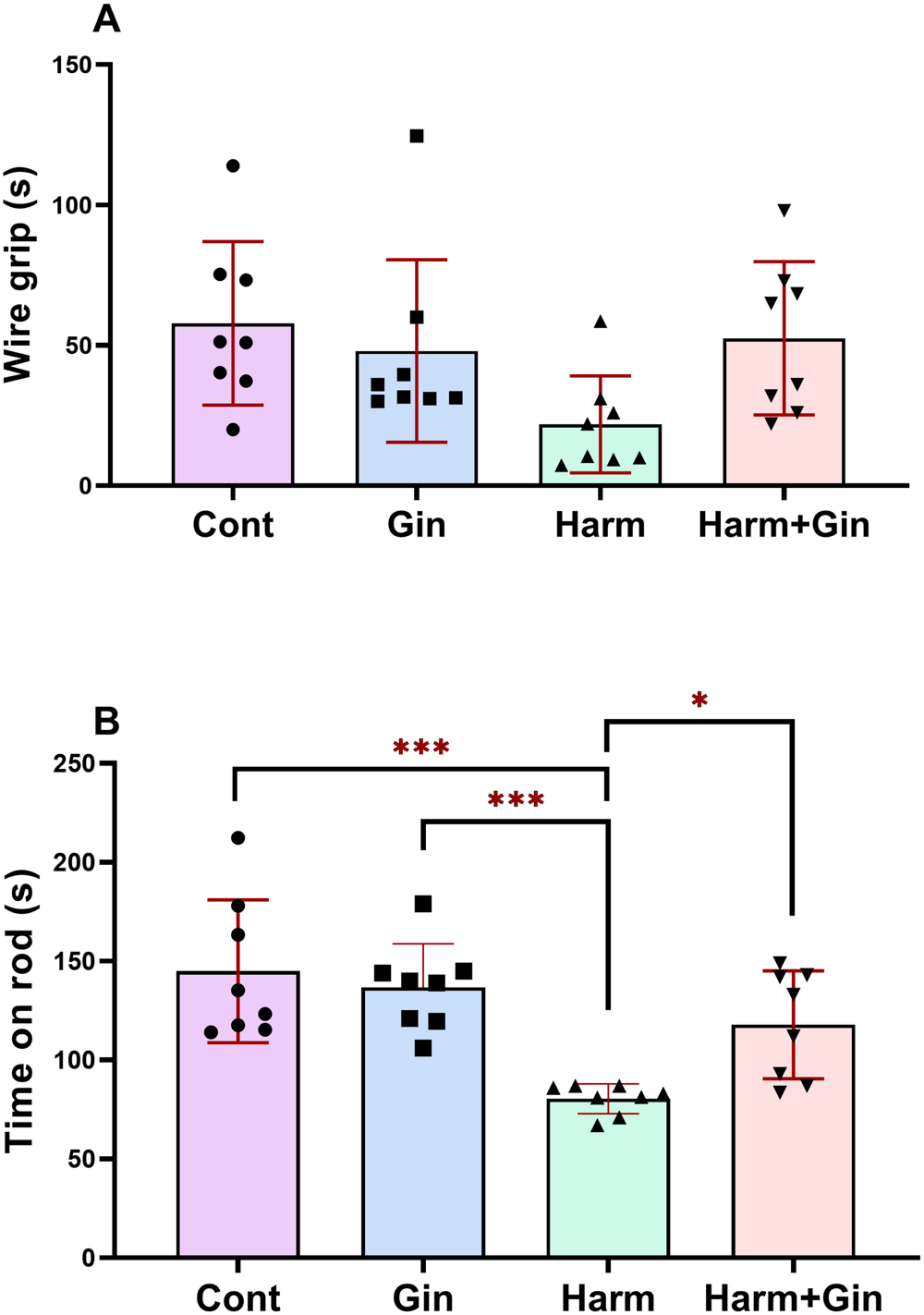
Assessment of motor coordination and muscle strength. (A) Wire grip test measuring grasping time and (B) rotarod test measuring latency to fall across experimental groups (*n* = 8 per group). Data are expressed as mean ± SEM. *p < 0.05, ***p < 0.001. Cont, control group; Gin, Ginseng group; Harm, Harmaline group; Harm + Gin, Harmaline + Ginseng group.

In the wire grip test, although there appeared to be a trend toward reduced performance in the Harmaline group, no statistically significant differences were observed between groups (Figure 4A). However, rotarod performance showed significant impairment in the Harmaline group compared to both control and Ginseng groups (*p* < 0.001) (Figure 4B). Korean red Ginseng treatment improved this deficit, as evidenced by significantly increased time on the rod in the Harmaline + Ginseng group compared to the Harmaline group (*p* < 0.05) (Figure 4B).

### 3.4 Gait Parameters Remain Unaffected in Harmaline‐Induced ET Model

**FIGURE 5:**
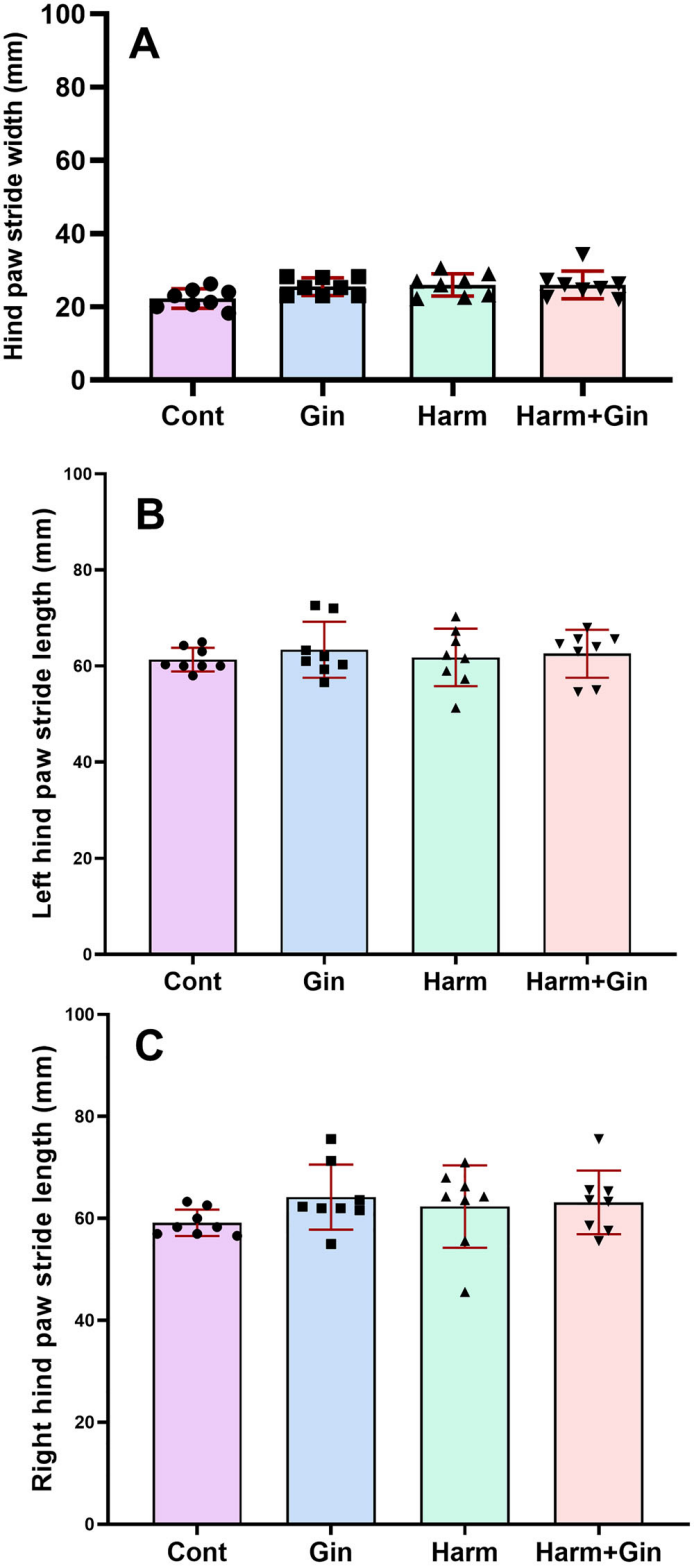
Assessment of gait parameters using footprint analysis. (A) Hind paw stride width, (B) left hind paw stride length, and (C) right hind paw stride length measurements across experimental groups. Data are expressed as mean ± SEM. Cont, control group; Gin, Ginseng group; Harm, Harmaline group; Harm + Gin, Harmaline + Ginseng group.

Footprint analysis revealed no significant differences in gait parameters across experimental groups. Neither stride width (Figure 5A), nor right stride length (Figure 5B) and left stride length (Figure 5C) showed statistically significant alterations in Harmaline‐treated animals compared to control subjects. Furthermore, Korean red Ginseng treatment had no significant effect on these gait parameters.

### 3.5 Korean Red Ginseng Attenuates Anxiety‐Like Behaviors in Harmaline‐Treated Mice

**FIGURE 6:**
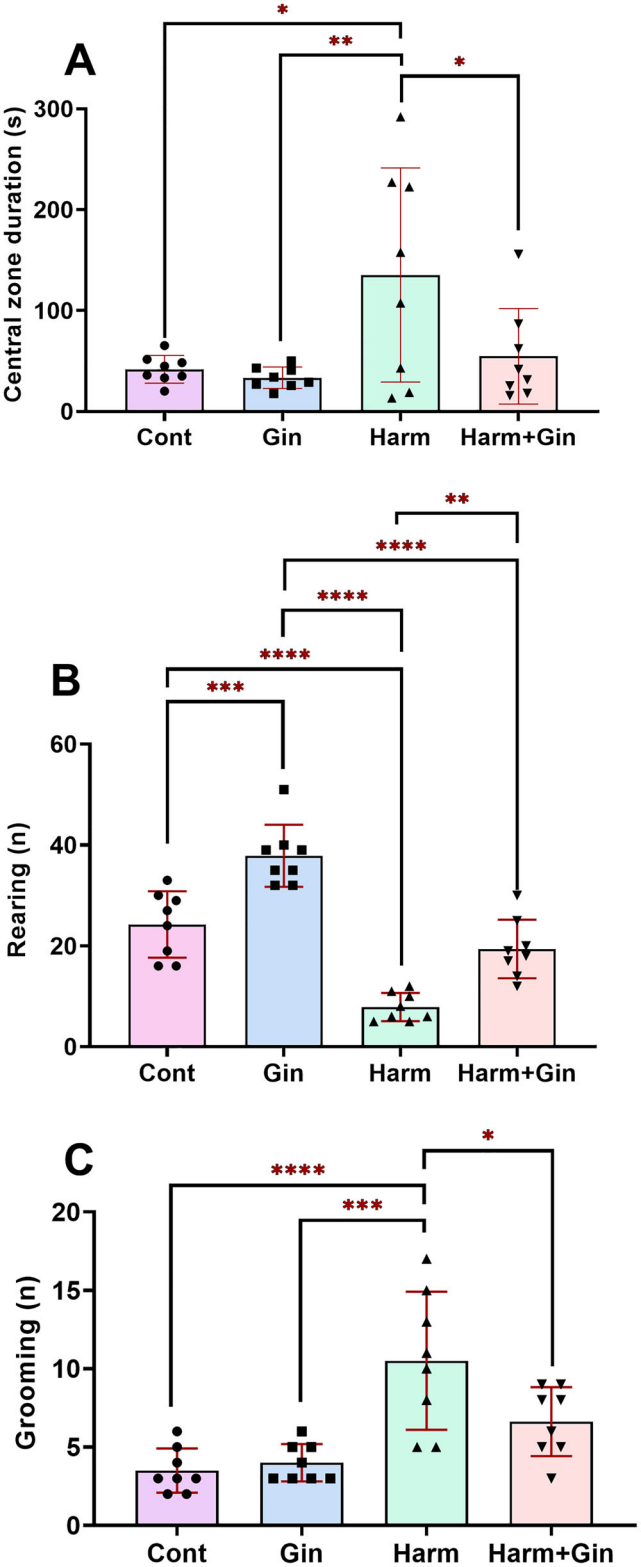
Anxiety‐like behaviors measured in the open field test. (A) Central zone duration, (B) rearing frequency, and (C) grooming frequency. Data are presented as mean ± SEM. *p < 0.05, **p < 0.01, ***p < 0.001, ****p < 0.0001. Cont, control group; Gin, Ginseng group; Harm, Harmaline group; Harm + Gin, Harmaline + Ginseng group.

Analysis of anxiety‐related parameters in the open field test revealed that the Harmaline group showed significantly increased time spent in the central zone compared to control (*p* < 0.05) and Ginseng (*p* < 0.01) groups. Notably, Korean red Ginseng co‐treatment significantly reversed this effect (*p* < 0.05) (Figure 6A). Additionally, rearing behavior was significantly reduced in the Harmaline group compared to control (*p* < 0.0001) and Ginseng (*p* < 0.0001) groups. Korean red Ginseng co‐treatment effectively normalized the Harmaline‐induced alterations in rearing behavior (*p* < 0.01) (Figure 6B). Grooming frequency was also significantly elevated in the Harmaline group compared to control (*p* < 0.0001) and Ginseng (*p* < 0.001) groups. Similarly, co‐treatment with Ginseng reversed this deficit (*p* < 0.05) (Figure 6C).

**FIGURE 7:**
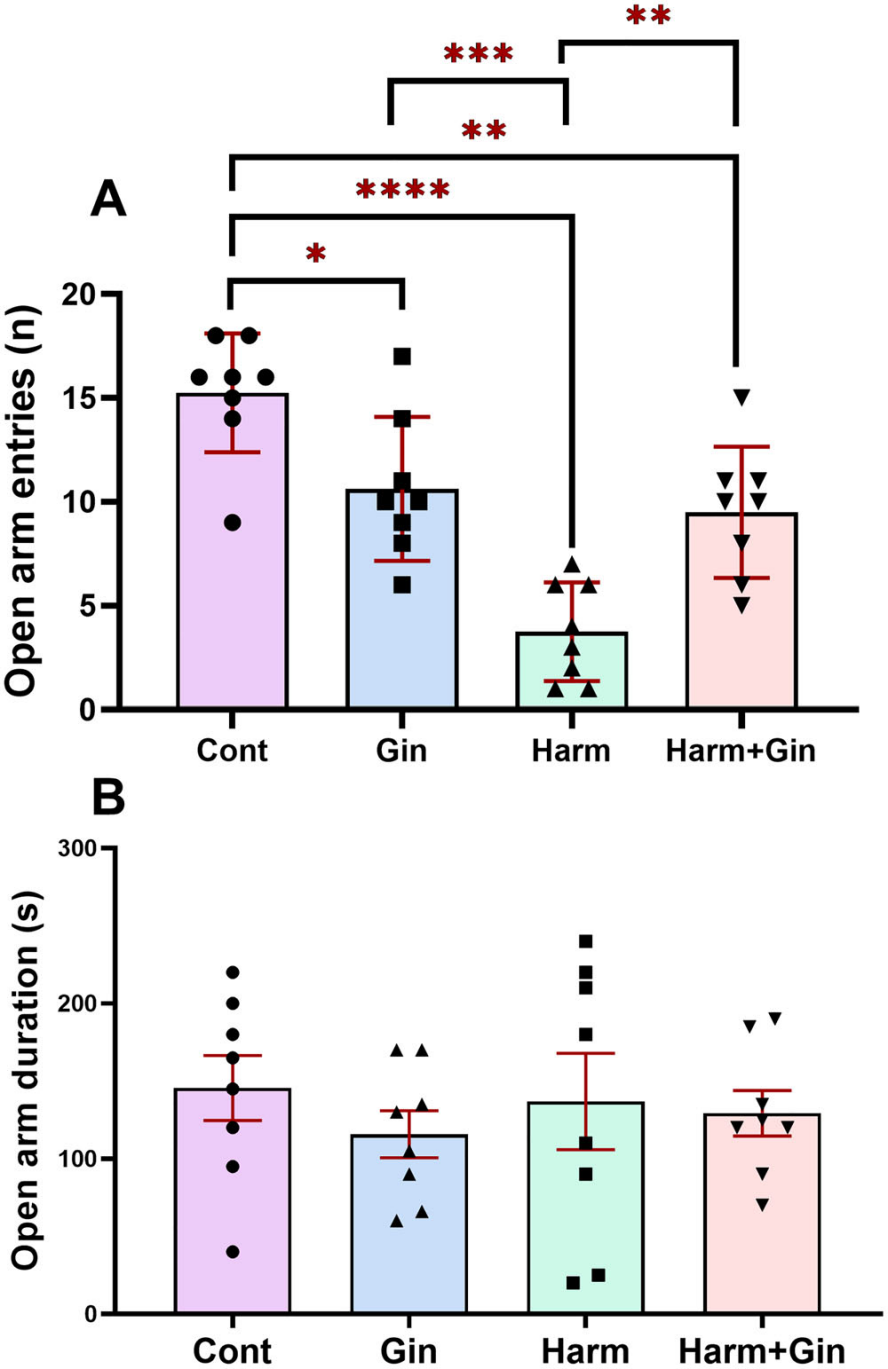
Assessment of anxiety‐like behaviors using the elevated plus maze. (A) Number of open arm entries and (B) time spent in open arms. Data are presented as mean ± SEM. *p < 0.05, **p < 0.01, ***p < 0.001, ****p < 0.0001. Cont, control group; Gin, Ginseng group; Harm, Harmaline group; Harm + Gin, Harmaline + Ginseng group.

A more specific analysis of anxiety‐like behaviors using the elevated plus maze testing showed that the Harmaline group had significantly fewer open arm entries compared to the control (*p* < 0.0001) and Ginseng (*p* < 0.01) groups. Notably, Korean red Ginseng co‐treatment significantly reversed this effect (*p* < 0.001) (Figure 7A). The time spent in open arms showed no significant differences between groups (Figure 7B).

### 3.6 Effects of Korean Red Ginseng on Depression‐Like Behavior in Harmaline‐Treated Mice

**FIGURE 8:**
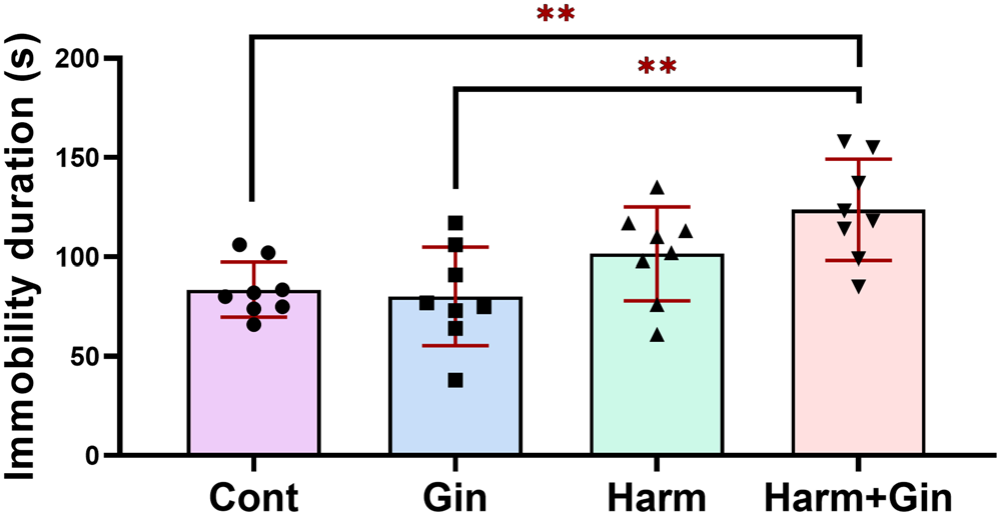
Assessment of depressive‐like behavior using the tail suspension test. Immobility duration across experimental groups. Data are expressed as mean ± SEM. **p < 0.01. Cont, control group; Gin, Ginseng group; Harm, Harmaline group; Harm + Gin, Harmaline + Ginseng group.

Analysis of depressive‐like behavior in the tail suspension test revealed that the Harmaline group did not show differences in immobility duration compared to control and Ginseng groups. Notably, no difference was seen between Ginseng‐only and control groups. However, higher immobility duration was observed in the Harmaline + Ginseng group compared to control and Ginseng groups (*p* < 0.01) (Figure 8).

### 3.7 Korean Red Ginseng Prevents Harmaline‐Induced Deficits in Passive Avoidance Learning

**FIGURE 9:**
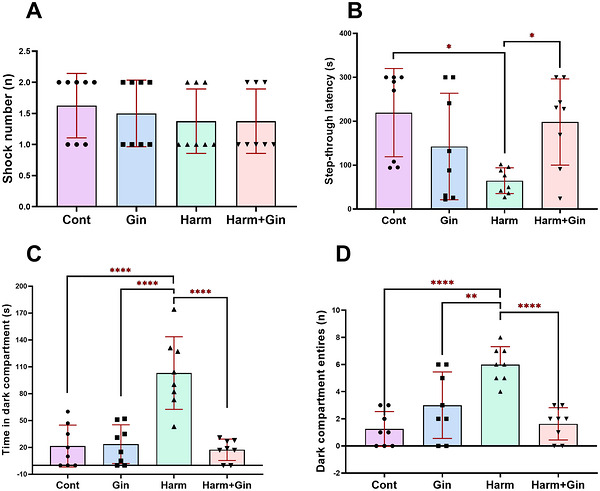
Assessment of learning and memory using a passive avoidance test. (A) Number of shocks required for learning, (B) step‐through latency, (C) time spent in dark compartment, and (D) number of dark compartment entries across experimental groups. Data are expressed as mean ± SEM. *p < 0.05, **p < 0.01, ****p < 0.0001. Cont, control group; Gin, Ginseng group; Harm, Harmaline group; Harm + Gin, Harmaline + Ginseng group.

Analysis of passive avoidance test showed no differences in the number of shocks required for learning across all experimental groups (Figure 9A). However, the Harmaline group demonstrated significantly reduced step‐through latency compared to the control group (*p* < 0.05), which was reversed by Korean red Ginseng co‐treatment (*p* < 0.05) (Figure 9B). Moreover, time spent in the dark compartment was significantly increased in the Harmaline group compared to control and Ginseng groups (*p* < 0.0001), and this was reversed by Korean red Ginseng co‐treatment (*p* < 0.0001) (Figure 9C). Similarly, the number of dark compartment entries was significantly elevated in the Harmaline group compared to control (*p* < 0.0001) and Ginseng (*p* < 0.01) groups, and Korean red ginseng co‐treatment effectively reversed this effect (*p* < 0.0001) (Figure 9D).

### 3.8 Korean Red Ginseng Suppresses Harmaline‐Induced Inflammatory Response and Normalizes Neuroplasticity Gene Expression in the Cerebellum

**FIGURE 10:**
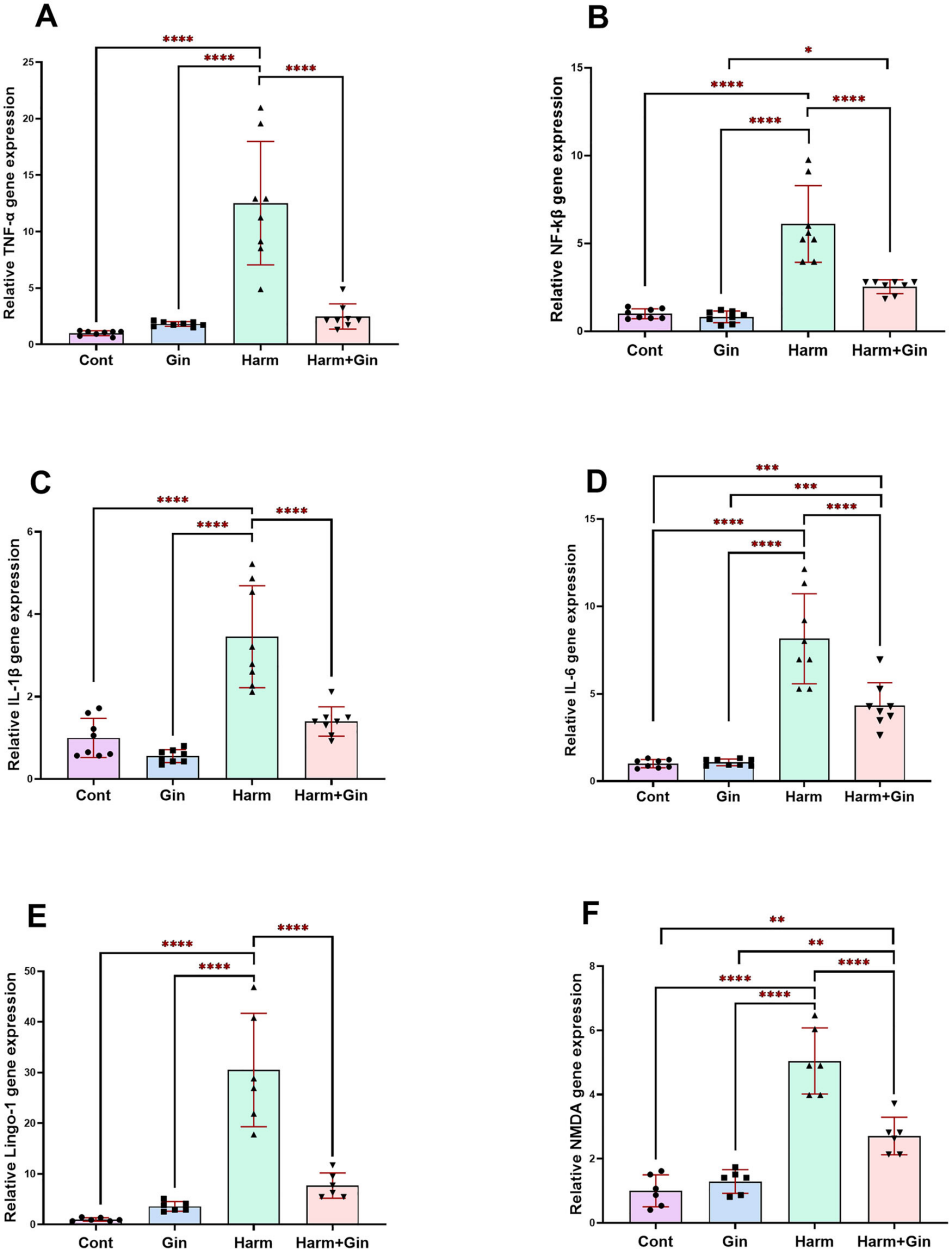
Assessment of gene expression in cerebellar tissue. Relative expression of (A) TNF‐α, (B) NF‐κβ, (C) IL‐1β, (D) IL‐6, (E) Lingo‐1, and (F) NMDA genes across experimental groups. Data are expressed as mean ± SEM. *p < 0.05, **p < 0.01, ***p < 0.001, ****p < 0.0001. Cont, control group; Gin, Ginseng group; Harm, Harmaline group; Harm + Gin, Harmaline + Ginseng group.

Analysis of inflammatory markers revealed that Harmaline significantly increased the expression of TNF‐α, NF‐κβ, IL‐1β, and IL‐6 compared to control and Ginseng groups (*p* < 0.0001) (Figure 10 A–D). Korean red Ginseng co‐treatment attenuated the Harmaline‐induced elevations in inflammatory markers, with significant reductions observed in TNF‐α, IL‐1β, NF‐kB and IL‐6 (*p* < 0.0001) (Figure 10 A–D).

Examination of neuroplasticity‐related genes showed that Harmaline significantly increased both Lingo‐1 and NMDA expression compared to the control and Ginseng groups (*p* < 0.0001) (Figure 10E,F). This upregulation was significantly reduced by Korean red Ginseng co‐treatment (*p* < 0.0001) (Figure 10E,F).

## 4 Discussion

The present study demonstrates that Korean red Ginseng exhibits significant therapeutic potential in a Harmaline‐induced model of ET in male mice, with benefits extending beyond tremor reduction to encompass improvements in motor coordination, anxiety‐like behaviors, and fear‐associated memory. Furthermore, our molecular analyses reveal that these behavioral improvements are accompanied by suppression of neuroinflammatory markers and normalization of neuroplasticity‐related gene expression in the cerebellum.

While Korean red Ginseng did not improve total distance moved, velocity, or mobility duration in open field testing, it significantly attenuated Harmaline‐induced tremor severity, which was an effect particularly evident on Days 3 and 5 of co‐treatment. Additionally, Korean red Ginseng co‐treatment effectively ameliorated disrupted motor coordination as evidenced by improvements in rotarod performance. These effects align with previous studies demonstrating the neuroprotective properties of Ginseng in various movement disorders. For instance, in a 3‐nitropropionic acid‐induced model of Huntington's disease, pre‐administration of Korean red Ginseng significantly attenuated neurological impairments and suppressed Huntington's‐like manifestations (Jang et al. 2013). Several preclinical studies have also shown the neuroprotective effects of Korean red Ginseng in Parkinson's disease (Choi et al. 2018; Jun et al. 2015). Interestingly, the therapeutic benefits of Korean red Ginseng have been reported in two patients, one with multisystem atrophy and another with spinocerebellar ataxia. As both patients demonstrated cerebellar atrophic changes, the report provides preliminary clinical evidence of potential disease‐modifying effects of Ginseng in cerebellar degeneration (Oh et al. 2015).

In our study, we also found an anxiolytic effect of Korean red Ginseng in Harmaline‐treated mice as we saw normalization of anxiety‐related behaviors in both open field and elevated plus maze tests. Interpretation of anxiety‐like behavior in the present study requires careful consideration of the methodological characteristics and limitations of each behavioral assay. The open field test provides a composite measure of locomotor activity, exploratory behavior, and anxiety‐related responses, whereas the elevated plus maze is considered a more specific and ethologically valid assay for assessing anxiety‐like behavior (Carola et al. 2002; Walf and Frye 2007). In the present study, armaline‐treated mice exhibited reduced rearing and increased grooming behavior in the open field test, both of which are commonly associated with anxiogenic‐like states. Consistently, elevated plus maze testing revealed an anxiogenic‐like profile, as evidenced by significantly fewer open arm entries.

We interpret open field test center‐related measures primarily in the context of altered motor and exploratory behavior rather than as definitive indicators of anxiolytic or anxiogenic states. In contrast, elevated plus maze outcomes are prioritized as the more reliable measure of anxiety‐like behavior in this model. Notably, Korean red Ginseng treatment normalized elevated plus maze performance, supporting an anxiolytic‐like effect that is consistent with previous reports of Ginseng‐mediated modulation of stress and anxiety‐related behaviors (Yoon et al. 2023; Zhao et al. 2014). This anxiolytic effect of Korean red Ginseng is especially important given the high comorbidity of anxiety in ET patients, which often exacerbates tremor severity and impacts quality of life (Chandran and Pal 2012; Sengul et al. 2015).

Clinical studies indicate that ET patients have higher anxiety scores than their age‐ and sex‐matched controls (Acar and Acar 2018). Neuroimaging findings further suggest that individuals with ET often exhibit depression and anxiety which are associated with structural brain changes (Sengul et al. 2019). The anxiolytic effects of Korean red Ginseng observed in our study are consistent with its documented anti‐anxiety properties across various experimental models. Previous studies have demonstrated that Korean red Ginseng effectively reduces anxiety‐like behaviors in multiple contexts, including during ethanol withdrawal (Zhao et al. 2014), post‐traumatic stress disorder models following single prolonged stress (Lee et al. 2020), and immobilization‐induced stress (Bui et al. 2022). The ability of Korean red Ginseng to ameliorate anxiety‐like behaviors while simultaneously reducing tremor suggests a potential dual therapeutic benefit in ET that could be particularly valuable in clinical applications.

Our findings in the passive avoidance test showed that Korean red Ginseng administration significantly restored fear‐associated memory, highlighting its protective effects against deficits in aversive associative memory that can be associated with ET. ET involves disruptions in cortical–cerebellar networks that can influence cognitive functions, particularly verbal working memory (Passamonti et al. 2011). Cerebellar neurodegeneration in ET appears to impair the communication between brain regions crucial for working memory and executive function (Passamonti et al. 2011). Interestingly, despite distinct pathophysiological origins, ET patients show memory impairment patterns similar to those observed in Parkinson's disease, particularly in tasks requiring greater executive organization, which suggests that disruption of thalamo‐frontal circuitry may represent a common pathway leading to specific cognitive deficits in both disorders (Lafo et al. 2015). Recent studies have also demonstrated subclinical memory impartment in ET patients which is associated with hippocampal microstructural damage (Novellino et al. 2020). Therefore, approaches to prevent cognitive impairments in ET are highly relevant (Janicki et al. 2013), and our data and others suggest that Korean red Ginseng could be therapeutic.

Long‐term administration of Korean red Ginseng has been shown to ameliorate age‐related decline in learning and memory (Lee and Oh 2015). Moreover, in a very recent study, administration of Korean red Ginseng effectively improved memory and cognitive behavior in 18‐month old mice (Shin et al. 2025). Furthermore, Korean red Ginseng was shown to attenuate cognitive impairments in animal model of alcohol‐induced memory deficits (Kim et al. 2023) and improve memory dysfunctions induced by scopolamine (Kang et al. 2013). Notably, studies have shown that the ability of Korean red Ginseng to enhance learning and memory is associated with hippocampal neurogenesis (Ryu et al. 2020). Taken together, Korean red Ginseng's demonstrated ability to enhance cognitive function across various experimental models, combined with its protective effects against ET‐related motor symptoms, makes it a particularly promising therapeutic candidate for addressing both the motor and cognitive aspects of ET.

An unexpected finding of our study was the increased immobility time observed in the tail suspension test in the Harmaline + Ginseng group, despite the absence of such an effect in either Harmaline‐ or Ginseng‐only groups. This result requires careful interpretation. As extensively discussed, immobility in the tail suspension test should not be viewed as a direct surrogate of depressive‐like behavior, but rather as a behavioral output reflecting stress‐coping strategies and the balance between active escape‐directed behavior and passive disengagement in response to an inescapable stressor (Cryan et al. 2005). Within this conceptual framework, immobility may represent an adaptive, energy‐conserving coping strategy rather than “behavioral despair,” particularly when considered alongside other behavioral domains. Notably, the increased immobility in our study occurred in the context of reduced anxiety‐like behavior, improved motor coordination, preserved learning and memory, and suppressed neuroinflammatory signaling, a behavioral profile that is not consistent with an overall depressive effect. Importantly, Ginseng alone did not increase immobility, suggesting that this effect is context‐dependent rather than an intrinsic adverse effect of the compound. Therefore, this finding may reflect a shift toward a more passive or energy‐conserving coping strategy under acute stress conditions. Nevertheless, given the inherent limitations of the tail suspension test, alternative interpretations of increased immobility, including a potential subtle depressive‐like effect, cannot be entirely excluded, although such an explanation appears unlikely when considered in the context of the broader behavioral and molecular findings. Accordingly, future studies should validate this finding using additional affective behavioral assays.

Our study revealed several potential mechanisms that can contribute to the therapeutic effects of Korean red Ginseng. The significant reduction in cerebellar expression of pro‐inflammatory markers (TNF‐α, NF‐κB, IL‐1β, and IL‐6) suggests that anti‐inflammatory actions could contribute to symptomatic benefits and potential long‐term neuroprotective properties. This finding is particularly relevant given the growing evidence implicating neuroinflammation in ET pathogenesis (Aygün and Dundar 2024; Muruzheva, Ivleva, et al. 2022; Muruzheva, Traktirov, et al. 2022). Our data showing that Korean red Ginseng suppresses multiple inflammatory mediators suggests a broad anti‐inflammatory effect that could contribute to symptomatic benefits and potential long‐term neuroprotective properties. The derived components of Korean red Ginseng have demonstrated significant anti‐inflammatory effects both in vitro and in vivo (Baek et al. 2016). In addition, long‐term administration of Korean red Ginseng water extract has been shown to effectively suppress the expression of multiple pro‐inflammatory genes including IL‐1β, IL‐8, TNF‐α, and IL‐6 across various organs in aged mice (Kim et al. 2021). Other studies suggest that Korean red Ginseng exerts its anti‐inflammatory effects through inhibition of NF‐κB and activating protein‐1 (AP‐1) signaling pathways in macrophages during inflammatory responses (Lee et al. 2022). Together, these findings indicate that the anti‐inflammatory properties of Korean red Ginseng may contribute to its neuroprotective properties.

Finally, our data suggest that Korean red Ginseng may improve motor function and fear‐associated memory in the harmaline ET model which is accompanied by normalizing Lingo‐1 levels and reducing NMDA receptor overexpression, which could be key mechanisms in its effect. Elevated cerebellar Lingo‐1 expression has been associated with ET pathogenesis (Delay et al. 2014), and in support of a role of Lingo‐1 in ET, in two of our previous investigations, we reported the overexpression of Lingo‐1 in the Harmaline‐induced model of ET (Pirmoradi, Esmaili, et al. 2025; Pirmoradi et al. 2024). As a negative regulator of oligodendrocyte development and myelin formation, Lingo‐1 has emerged as a potential therapeutic target in ET, with studies showing that blocking its activity can promote remyelination processes (Agúndez et al. 2015). Thus, the normalization of Lingo‐1 overexpression by Korean red Ginseng treatment in our study may contribute to improved neuronal function and survival.

Similarly, the decrease in NMDA receptor overexpression by Korean red Ginseng treatment also suggests potential effects on synaptic plasticity and neurotransmission, which could contribute to the observed improvements in motor coordination and cognitive function. NMDA receptors are essential mediators of synaptic plasticity in the cerebellum, where they contribute to motor learning and coordination (Piochon et al. 2010). While NMDA receptors typically promote synaptic plasticity in most brain regions, Purkinje cell spines in the cerebellum naturally lack postsynaptic NMDA receptors at their parallel fiber inputs‐a specialization that appears crucial for motor learning (Galliano et al. 2018). In transgenic lines overexpressing NMDA receptors, NMDA receptor‐mediated currents at these synapses impaired long‐term potentiation and disrupted motor learning processes including vestibulo‐ocular reflex adaptation (Galliano et al. 2018). Therefore, our observation of an increased NMDA receptor expression in the Harmaline model may represent a pathological alteration that contributes to motor dysfunction, and Korean red Ginseng's ability to normalize NMDA receptor levels could help restore motor learning processes.

It should be noted that the overall gene expression changes observed in this study represent indirect proxies of underlying neurobiological processes and do not necessarily reflect functional alterations at the circuit level. Therefore, while these findings provide important mechanistic insights, further studies incorporating electrophysiological, protein‐level, or circuit‐based approaches are required to confirm their functional significance. Moreover, in interpreting these results, it is important to distinguish between symptomatic and mechanistic interpretations of these findings. The observed improvements in tremor severity, anxiety‐like behavior, and cognitive performance primarily reflect symptomatic modulation within the context of an acute/sub‐chronic pharmacological model. In contrast, the accompanying molecular changes, including alterations in neuroinflammatory and neuroplasticity‐related markers, may provide insight into underlying biological processes but do not, in themselves, establish definitive disease‐modifying effects.

From a translational perspective, the behavioral improvements observed in the present study are unlikely to arise from modulation of any single molecular target. Rather, they likely reflect coordinated changes across multiple levels of neurobiological organization, including inflammatory signaling, synaptic plasticity, and circuit‐level dynamics. Such a systems‐oriented view is increasingly recognized in neuroscience, where behavioral outputs emerge from the integrated activity of distributed networks rather than isolated pathways (Bargmann and Marder 2013; Guiral 2026). For example, recent studies have demonstrated that cognitive and behavioral functions such as cue‐guided behavior and action inhibition depend on interactions between higher‐order cognitive processes, synaptic plasticity mechanisms, and functional connectivity within neural circuits (Garofalo et al. 2019; Però et al. 2025). Within this framework, the effects of Korean red Ginseng observed here may be best understood as reflecting multilevel neuromodulation that collectively shapes motor, affective, and cognitive outcomes, rather than a unidimensional mechanistic action. Collectively, the results of this study reveal a convergent pattern across behavioral domains, whereby improvements in motor coordination are paralleled by normalization of anxiety‐like behavior and restoration of fear‐associated learning and memory, supporting the notion that Korean red Ginseng exerts coordinated modulatory effects on cerebellar circuits underlying both motor and nonmotor functions.

Several limitations of the current study should be acknowledged. First, while the Harmaline‐induced model is a well‐established and widely used paradigm for eliciting tremor and cerebellar circuit dysfunction relevant to ET, it represents an acute/sub‐chronic pharmacological model. As such, it does not fully capture the progressive and potentially neurodegenerative features of human ET, which typically evolve over years. Accordingly, the beneficial effects of Korean red Ginseng observed in this study should be interpreted primarily in terms of symptomatic relief and modulation of tremor‐associated inflammatory and neuroplasticity‐related pathways, rather than definitive modification of long‐term disease progression. Future investigations using chronic, progressive, or genetic models of ET will be necessary to determine whether Korean red Ginseng exerts sustained neuroprotective or disease‐modifying effects over extended time scales. Second, our study focused on relatively short‐term effects of Korean red Ginseng treatment, and longer‐term studies would be valuable to assess the durability of the observed benefits and potential development of tolerance.

Another limitation of this study was that we used a single dose of Korean red Ginseng (100 mg/kg), and dose‐response studies would be necessary to determine the optimal therapeutic window. In addition, the sample size used for molecular analyses (*n* = 4 per group) was relatively limited, which may reduce statistical power and increase susceptibility to variability in gene expression measurements. Therefore, these findings should be interpreted with caution and warrant replication in studies with larger sample sizes. Furthermore, all experiments in our study were conducted exclusively in male mice, and therefore the observed behavioral and molecular effects of Korean red Ginseng cannot be generalized to females. This limitation is important given the evidence of sex‐related differences in epidemiology, clinical presentation, and mechanistic pathways involved in ET (Arabia et al. 2022; Hubble et al. 1997). Moreover, ovarian hormones are known to modulate inflammatory cascades and synaptic plasticity (Hyer et al. 2018; Osborne et al. 2018), and may consequently alter the neurobiological response to both Harmaline exposure and Ginseng‐derived compounds. As a result, the magnitude, direction, or mechanisms of Korean red Ginseng's effects observed in male mice may differ in females and future studies incorporating both sexes and examining sex‐specific behavioral and molecular outcomes will be essential. Additionally, investigation of different preparations and extraction methods of Korean red Ginseng could provide insights into which components are most crucial for its therapeutic effects.

## 5 Conclusions

In conclusion, this study demonstrates that Korean red Ginseng ameliorated both motor and nonmotor symptoms in a Harmaline‐induced model of ET, including reduced tremor severity, improved motor coordination, and attenuated anxiety‐like behaviors and cognitive deficits. These therapeutic effects are accompanied by suppression of neuroinflammatory markers and reduction in Lingo‐1 and NMDA receptor gene expression in the cerebellum. Overall, these effects likely reflect symptomatic modulation and regulation of tremor‐related neuroinflammatory and neuroplasticity‐associated processes within an acute/sub‐chronic experimental context. While these results highlight the therapeutic potential of Korean red Ginseng, they should not be interpreted as evidence of definitive disease modification. Future studies employing chronic or progressive models will be necessary to determine whether these effects translate into sustained neuroprotective or disease‐modifying outcomes.

## Author Contributions

**Monavareh Soti**: conceptualization, methodology, investigation, writing – original draft, writing – review and editing. **Mehran Ilaghi**: conceptualization, investigation, writing – original draft. **Leila Bagherzadeh**: conceptualization, writing – original draft, writing – review and editing. **Fatemeh Shahsavari**: conceptualization, methodology, investigation, data curation. **Kristi A. Kohlmeier**: conceptualization, writing – review and editing, supervision. **Zeynab Pirmoradi**: conceptualization, methodology, formal analysis, supervision, project administration, writing – review and editing. **Mohammad Shabani**: conceptualization, formal analysis, resources, supervision, funding acquisition, writing – review and editing.

## Funding

Funding for this study was provided by Kerman University of Medical Sciences (grant no. 403000067).

## Ethics Statement

The experimental procedures were approved by the Ethics Committee of Kerman University of Medical Sciences under ethical code of IR.KMU.AEC.1403.038.

## Conflicts of Interest

The authors declare no conflicts of interest.

## Data Availability

Data from this study will be made available by the corresponding author upon request.
